# What can we learn from scoliosis in children with the 22q11.2 deletion syndrome? Prognostic factors at pre-adolescent age for fast progressive, mild and self-resolving forms during adolescence

**DOI:** 10.1007/s43390-025-01073-4

**Published:** 2025-03-20

**Authors:** Sabrina Donzelli, Peter Lafranca, Maarten Van Smeden, René Castelein, Tom Schlösser

**Affiliations:** 1 Istituto Auxologico Italiano, Milan, Italy; 2 Department of Orthopedic Surgery, UMC Utrecht, Utrecht, Netherlands; 3 Julius Center for Health Sciences and Primary Care, Utrecht, Netherlands

**Keywords:** Spine, Scoliosis, Deformity, Prediction, Screening

## Abstract

**Introduction:**

Longitudinal data starting before adolescence and before curve onset, may elucidate prognostic factors for later scoliotic curve development. The aim is to predict the maximum curve acceleration (MCA; °/month) and the final curve progression in a cohort of 22q11.2DS subjects screened for scoliosis.

**Methods:**

Scoliosis screening starts immediately after 22q11.2DS diagnosis. A minimum of 2 years follow-up, two assessments, Risser 0, open triradiate cartilage at start, were the inclusion criteria. Risser ≥ 3 corresponded to skeletally matured. Linear and logistic binary mixed effect models accounting for patients nested into multiple measurement occasions were created to predict MCA during adolescence and progressors (progression to ≥ 30) versus non-progressors (no scoliosis or < 30 at last follow-up).

**Results:**

161 subjects (59% females) with a mean baseline age 8.7 ± 2.4 years, follow-up of 4.2 ± 2.4 years and having reached skeletal maturity. Ultimately, 19 subjects became progressors and 142 became non-progressors. Curve magnitude at baseline was 8.8 ± 5.9° (range 0–50), at final follow-up 11.6 ± 12.4 (0–77). The mean curve acceleration was + 0.1 ± 0.5°, respectively + 0.2 ± 0.5°for non-progressors vs progressors during the acceleration phase. A linear mixed model showed that the triradiate cartilage closure accelerates MCA by 2.6 when adjusted for age and female gender. In a logistic mixed model, when the triradiate cartilage closes, the OR of reaching the MCA before the next follow-up is increased by 4.60 (CI95% 2.34–8.90 *p* < 0.001). No evidence for prognostic value of Risser in all derivated models.

**Conclusion:**

We found no evidence for the parameters in the coronal, sagittal nor transverse plane before curve onset acting as prognostic factors for curve behavior. In the prediction model on a longitudinal database that starts in many patients before scoliosis, no clear radiographic discriminant for later progressive scoliosis could be identified. The closure of the triradiate cartilage resulted as the best sign of pubertal spurt onset and scoliosis acceleration.

## Introduction

Adolescent idiopathic scoliosis (AIS) prevalence in the general population is around 1–3%, but in individuals with 22q11.2DS scoliosis development is much higher at around 50%. Despite obvious differences with AIS, previous studies have suggested that this specific syndromic population develops curves that share certain mechanistic characteristics with idiopathic scoliosis, and can be used as a human model to elucidate certain biomechanical factors in the onset and progression of scoliosis more in general [1, 2]. Many studies have investigated the natural history of AIS. Natural history is the evolution of a disease over time when untreated [3]. However, population-based studies have been performed only cross-sectionally and all longitudinal studies focus on patients with already established scoliosis and follow later curve progression. Understanding natural history helps to understand the pathogenesis and optimize the timing and impact of interventions for structural scoliosis, such as bracing, scoliosis-specific exercises or surgery (4). For true prevention or early treatment of scoliosis, monitoring of pre-clinical patients-at-risk is required to detect any initial omen of scoliosis development or progression (1).

Understanding the earliest natural history of scoliosis development is essential for development of curative or preventive treatments. For this, ideally, a model should enable physicians to identify patients exposed to higher risks as early as possible with the goal to provide the easier and less invasive, and most effective treatment. In the past, attempts for early detection have been made through school screening. However, the low prevalence of idiopathic scoliosis made school screening cost ineffective and furthermore, it led to overdiagnosis and overtreatment [5]. Models are widely used in biomedical research to better understand patho-mechanism and treatment consequences, knowing that the model only shares limited characteristics with the ‘real’ population. Zebra fish are widely used to study genetics in scoliosis, while they share few similarities with humans. Unfortunately, no true biomechanical model for scoliosis exists, because human bipedal spinal loading is unique even compared to non-human primates [6, 7]. Scoliosis in 22q11.2DS develops on an initially normal spine and shares certain characteristic with AIS that makes it an interesting model to study factors related to biomechanical spinal loading [2]. The risk of scoliosis development in 22q11.2DS patients is high and therefore routine radiographic screening is recommended. In our national 22q11.2DS referral center, we have implemented such a screening program with regular follow-up which starts, at syndrome diagnosis which is mostly before the growth spurt [8].

Longitudinal registry data of spinal growth, starting before adolescence, in this high-risk population may elucidate prognostic factors for later structural scoliotic curve development. The aim of this study is to investigate the discriminant factors at preadolescent age between higher risks forms compared to lower risk subjects and self-resolving curves. The longitudinal structure of the systematically collected data also offers the possibility to investigate which radiographic and demographic parameters can predict the final curve magnitude as a continuous variable and the timing of maximum curve acceleration (MCA, °/month).

## Materials and methods

### Study design and ethics

This study is designed as a retrospective cohort study of a population of subjects prospectively screened for scoliosis using clinical assessment and routine biplanar full-spine radiographs. The local Ethical Review Boards of the university hospital involved approved this study and waived the necessity of explicit (parental) informed consent since data were collected as part of standard care and were handled anonymously. Biplanar spinal radiographs are made of each patient at 2-year intervals as part of a global standard 22q11.2DS follow-up protocol, and more frequent in patients with proven scoliosis (9).

### Study population

**Fig. 1:**
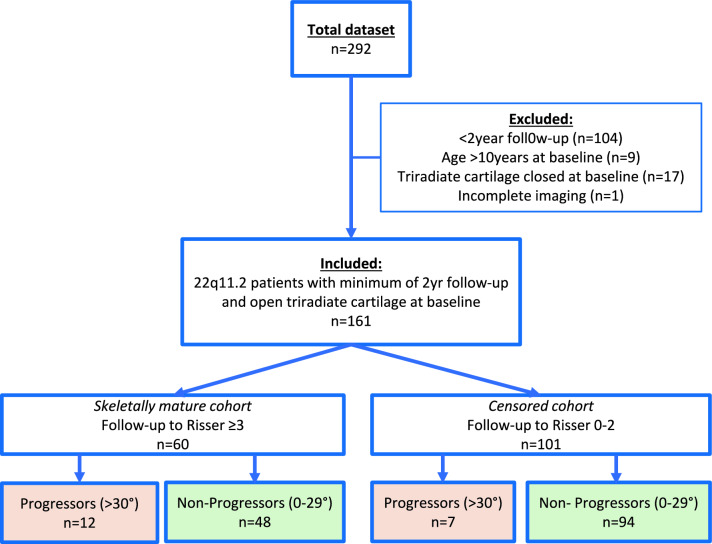
Flowchart of the selection process

From the database of the national 22q11.2DS clinic, all pediatric patients (0–18 years) that were ambulant, had a genetically confirmed 22q11.2 deletion or duplication, and underwent the systematic scoliosis screening were included. For inclusions, at least one radiograph should include Risser 0 with the triradiate cartilage open, and a follow-up assessment with a minimum of 2-year follow-up should be available. Non-ambulatory patients and patients with congenital scoliosis were excluded [9]. Sex, age at the time of radiography, and data on comorbidities were collected. The patients with missing x-rays preventing measurements were also excluded [9, 10]. Also, the cohort included subjects that had not yet reached the end of growth during the follow up. We called this cohort the ‘censored cohort’, because these subjects could not have shown scoliosis or maximum curve progression because the observation period ended too early (Fig. 1). Considering the limited available sample size, and to optimize the models developed, according to the results at the end of growth, patients were classified into two categories:

- Progressors:
  o Those subjects with curves > 30° at last follow-up.
- Non-progressors
  o No or minimal scoliosis: self-resolving or non-progressive forms, never exceeding 29°.

### Radiographic measures

All radiographic measurements were performed in Surgimap imaging software version 2.3.2.1 (Nemaris Inc., NY, USA) and in PACS (IDS7 version 23.1.10, Sectra AB, Linköping, Sweden). One experienced observer, blinded for all other clinical parameters, analyzed all radiographs in chronological order. On all posterior-anterior images available, the following parameters were measured:

- Triradiate cartilage closure
- Risser stage
- Curve magnitude angle of the major curve
- Monthly Cobb change and maximum curve acceleration (MCA) was calculated from the curve magnitude measured at each occasion divided by time in month between occasions. MCA happened generally once in each patient. To discriminate the real curve magnitude changes from the variation due to postural changes, positioning or other sources of measurement errors we defined a threshold: in patients with true scoliosis the maximum peak event happened when the MCA > 0.16°/month.
- Rotation of the apex at the major curve (Nash-Moe) at baseline
- C7-SVA in the coronal and sagittal plane at baseline

On the lateral radiographs at baseline, the following parameters were measured:

- The PIT-area, representing the posteriorly inclination of the thoracic and lumbar spine and posteriorly directed shear loads. The PIT-area was automatically calculated following the basic formula: 0.5 * width * height [21].

### Statistical analysis

Statistical analyses were performed using STATA 17.0 MP—Parallel Edition for Windows (Copyright 1985–2021 StataCorp LLC). Descriptive statistics included mean and standard deviation for continuous variables normally distributed. Proportion and rates have been used to describe categorical binary and multilevel variables. To check differences between the included cohorts (censored versus skeletal mature and progressors versus non-progressors) a t-test was run for continuous variables, alpha value was set below 0.05 to declare a statistically significant difference.

Mixed effect models have been chosen because of the advantages of taking into account the multiple measurement occasions structure of data and accounting for both the within and between subjects’ variability. All models have been tested first in the whole sample and then in the cohort reaching skeletal maturity. Linear models have been checked for linearity of the assumptions by checking residuals distribution in a histogram and through quantile plots. We pre specified prognostic factors, and check if they were practical, plausible and parsimonious. They have been tested in a univariable model and compared in the final multivariable mixed effect model. The outcome variable for the continuous model were curve magnitude in Cobb angle at measurement occasions. Outcome variable for the binary logistic model was the timing of MCA. Predictors: age was the time variable considered in the model. The other tested predictors were gender, the closure of triradiate cartilage, Risser stage and Cobb angle. The following baseline radiographic parameters have been tested only in the end of growth sample: the PIT-area, the apex vertebral rotation, the C7-SVA.

## Results

161 subjects were included. 60 of those were followed for an average of 3.2 ± 1.7 years and had reached skeletal maturity during the follow up period. 101 did not reach skeletal maturity, they were followed for an average of 6.0 ± 2.2 years. The gender distribution accounted for 59% of females. 73% of the population was aged 2 to 9 at baseline. The follow-up intervals were not consistent due to variation in age of diagnosis of scoliosis. The censored cohort included younger subjects, while the distribution of age in the skeletally mature cohort was more balanced between progressors and non-progressors (Table 1).

**Table 1:** Demographic summary

	All	Skeletal mature cohort	Censored cohort	*P* value
Age at baseline	8.7(2.4)	12.6(2.4	9.9(2.0)	0.000*
Age at follow-up	12.9(3.0)	16.0(1.7)	11.1(2.0)	0.000*
Female	59%	52%	63%	
Number of visits (2, 3,–≥ 4)	2 VIS = 61 3 VIS = 51 > 4 VIS = 49			
Final Risser stage	57% R0 0% R1 6% R2 10% R3 15% R4 12% R5	27% R3 40% R4 33%R5	90% R0 1%R1 9%R2	
Curve magnitude at baseline	8.8(6.95)	9.1(6.4)	8.4(2.0)	0.0235*
Curve magnitude at follow-up	11.6(12.4)	15.1(14.4)	9.5(10.5)	0.0062*
Progressors	19	12	7	
Monthly Cobb change(°/month)	0.06 (0.42)	0.05(0.27)	0.1(0.54)	0.0058*
Maximum Curve acceleration (°/month)	0.2(0.5)	0.32(0.38)	0.08(0.50(	0.0000*

Upper part all sample summary, lower part differences between censored and end of growth cohorts

*T-test alpha value below 0.05 showing statistically significant difference

Ultimately, 19 became a progressors, and 142 a non-progressors, 12 (20%) of the 60 patients in the skeletally mature cohort and 7 (7%) of the 101 in the censored cohort. Progressors had higher curve magnitude at start than milder forms (9.7° *versus* 6.6°, respectively, not significantly different, *P* = 0.1139) but still below the diagnosis threshold, thus confirming the follow up started early, before scoliosis onset or faster progression. Comparison of baseline parameters between progressors and non-progressors are shown in Table 2.

**Table 2:** Demographic comparison of the distribution of progressors in the two cohorts: censored and skeletally mature

	Skeletal mature cohort		*P* value	Censored cohort		*P* value
	Progressors 12 (20%)	Non-progressors 48 (80%)		Progressors 7(7%)	Non-progressors 94(93%)	
Age at baseline	11.3(2.2)	12.9(2.4)	0.3663	11.1(2.6)	9.8(1.95)	0.0008*
Age at follow-up	16.2(1.9)	15.8(1.6)	0.4825	13.1(1.7)	1.09(2.0)	0.0055*
Female	50%	52%		43%	65%	
Final Risser stage	33% R3 50% R4 17% R5	25% R3 37.5% R4 37.5% R5		71% R0 29% R2	92% R0 1% R1 7% R2	
Curve magnitude at baseline	14.8(6.0)	7.6 (5.7)	0.0354*	26.6(16.5)	7.0(5.3)	0.0000*
Curve magnitude at follow-up	34.9(20.0)	10.1 (6.5)	0.0000*	35.9(23.8)	7.6(5.1)	0.0000*
Monthly Cobb change(°/month)	0.30(0.66)	0.04 (0.21)	0.0001*	0.47(1.05)	0.09(0.38)	0.0000*
Maximum Curve acceleration (°/month)	0.96(0.40)	0.20 (0.16)	0.0000*	1.19(1.04)	0.06(0.32)	0.0000*

*T-test alpha value below 0.05 showing statistically significant difference

The MCA has been calculated in 41 subjects, including 17 progressors. The MCA was faster when the scoliosis became more severe and reached the maximum values in progressors. This is more evident if we compare the progressors to the non-progressors (Table 2).

### Full sample regression model

We first ran a mixed linear multilevel model for curve magnitude over time as a continuous outcome, in the whole cohort thus including skeletally mature as well as censored patients, with age as the time variable (see Table 3). The age is influencing the outcome because in practice, patients with more severe curves have been followed up more frequently. The linear mixed effect model showed that after the closure of the triradiate cartilage the curve magnitude progression is increased by 2.63 (CI95% 1.28–3.97 *p* = 0.001), adjusted by age (with a coefficient of 0.30 CI95% −0.06–0.76 *p* = 0.092), and being a male is protective from curve magnitude progression (female coefficient was − 1.66 95% CI − 3.5–0.19 *p* = 0.078).

**Table 3:** Mixed effect multilevel model predicting the curve magnitude over time, in the full cohort (including censored) and in the skeletal mature cohort

Full sample cohort model						
Mixed effect model predicting the curve magnitude over time	Unadjusted coefficient	CI 95%	*P* value	Adjusted coefficient	CI 95%	*P* value
Age	0.62*	0.23–1.02*	0.002*	0.35	− 0.06 to 0.76*	0.092
Triradiate closure	2.71*	1.36–4.04*	0.000*	2.63*	1.28–3.97*	0.001*
Female gender	− 1.90*	− 3.74 to 0.06*	0.043*	− 1.66*	− 3.50 to 0.19*	0.078*
Skeletal mature cohort model						
Age	1.30*	0.65–1.95*	0.000*	0.88	0.69–1.12	0.294
Triradiate closure	2.54*	0.85–4.22*	0.003*	2.96*	1.19–4.73*	0.001*
Female gender	− 3.92*	− 6.85 to 0.99*	0.009*	− 3.68*	− 6.67 to 0.70*	0.02*
Vertebral rotation at start	− 0.03	− 0.59 to 0 .53	0.925	0.15	− 0.37 to 8.56*	0.580

Adjusted and unadjusted coefficients are displayed in the table

*alpha value below 0.05 showing statistically significant difference

**Fig. 2:**
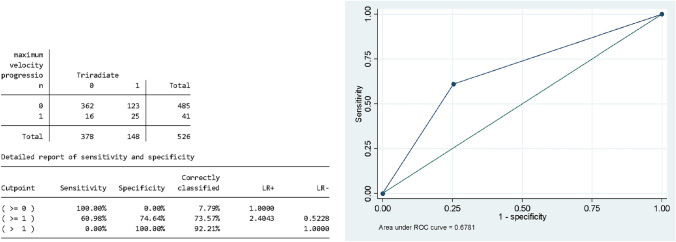
ROC Curve and the AUROC of full sample regression model

A logistic mixed effect multilevel model was run considering the monthly Cobb angle change between visits to identify factors for MCA. When the triradiate cartilage closes, the unadjusted OR of reaching the MCA is increased by 4.6 (CI95% 2.34–8.90 *p* < 0.001). There was no evidence for prognostic value of Risser stage or gender in all derivated models after adjustments for all the covariates. In the logistic model, age did not influence the odds of the maximum acceleration event; this confirms the low variance found in the linear model within individuals between occasions. Figure 2 is showing the ROC Curve and the AUROC of 0.68 (95% CI 0.60–0.76) of the TRC closure to predict the event of MCA (Table 3).

### Regression model in skeletally mature cohort

**Fig. 3:**
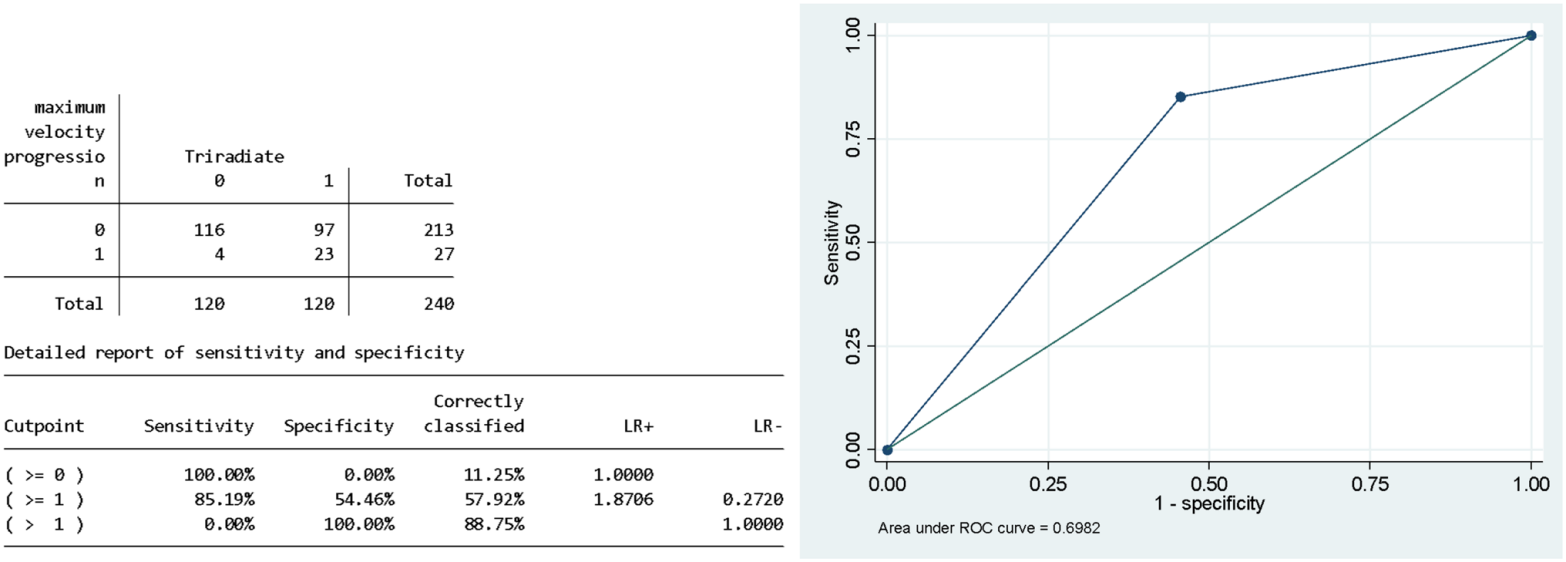
ROC Curve and the AUROC of the regression model in the skeletally mature sample

To test predictors in patients with an almost complete scoliosis history, a linear mixed multilevel model was applied to the parameters of the skeletally mature cohort (Table 4). When the triradiate cartilage closes the curve magnitude progression is increased by 2.96 (CI95% 1.19–4.73 *p* = 0.001) adjusted by age (coeff. = 0.88 CI95% 0.69–1.12, *p* = 0.294), and being a male is protective from curve magnitude progression (female coeff. = − 3.68 95% CI − 6.67–0.70 *p* = 0.02). The odds of reaching the MCA were significantly higher after the triradiate cartilage closure in both univariate and multivariate model and the prediction accuracy reached 70%: the OR of reaching the MCA at next follow-up adjusted by age (OR = 0.88 CI 95% 0.69–1.12 *p* = 0.294) female gender (OR 0.45 CI 95% 0.18–1.09 *p* = 0.078) and baseline vertebral rotation (OR 0.98 CI 95% 0.84–1.14 *p* = 0.820) (Table 4). The ROC curve and AUROC available in Fig. 3.

**Table 4:** Logistic Mixed effect multilevel model predicting the maximum Cobb acceleration in the full cohort (including censored) and in the skeletal mature cohort

Full sample cohort model						
Mixed effect model predicting the maximum Cobb velocity	Unadjusted OR	CI 95%	*P* value	Adjusted OR	CI 95%	*P* value
Age	1.29*	1.15–1.43*	0.000*	1.15	0.98–1.36	0.096
Triradiate closure	4.60*	2.38–8.90*	0.000*	2.19	0.82–5.82	0.118
Female gender	0.60	0.31–1.13	0.113	0 .61	0.31 1.20	0.153
Progressors	3.15*	1.62–6.11*	0.001*	2.34*	1.17–4.71*	0.017*

Skeletal mature cohort model						
Logistic model predicting the MCA	Unadjusted OR	CI95%	P value	Adjusted OR	CI95%	P value
Age	1.19	1.02–1.38	0.025	0.88	0.69- 1.12	0.294
Triradiate closure	6.88*	2.30–20.6*	0.001*	8.92*	2.57- 30.97*	0.001*
Female gender	0.55	0.25–1.25	0.154	0.45	0.18- 1.09	0.078
Vertebral rotation at start	1.00	0.86–1.16	0.189	0.98	0.84- 1.14	0.820

Adjusted and unadjusted OR are displayed in the table. MCA = Maximum Curve Acceleration

*alpha value below 0.05 showing statistically significant difference

## Discussion

Scoliosis can be more or less progressive and experts agree that scoliosis heterogeneous presentation could be roughly classified into milder curves without clinical consequences and more aggressive forms with impact for later quality of life. The earlier we can discriminate those exposed to higher risk, the more treatment options we have to offer to the patients and the fewer low-risk patients need to be followed.

Multiple attempts have been undertaken to find the predictors of severe scoliosis progression, most of the time in adolescents with already established idiopathic scoliosis, either by clinical symptoms of scoliosis, or as occasional radiographic findings. Prediction based on the precursor stage of scoliosis, before its onset, has not been performed to date.

In this prediction model on a longitudinal database that starts in many patients before scoliosis, no clear radiographic discriminant for later progressive scoliosis could be identified. Therefore, before scoliosis diagnosis, the subjects can be categorized as healthy and it is very difficult to find some omen of the pathology in the earliest phase, before spine deformity onset. Also, analysis of prognostic factors in the transverse and sagittal plane in the smaller sample that had reached the end of growth, did not demonstrate a good predictor of curve magnitude progression (11–14). Despite the early screening, it was not possible to find any radiographical sign of a progressive curve. Apparently, before scoliosis onset the patients had similar radiographic parameters in all planes. Interestingly, at prepubertal age, spinal asymmetries below the 10 degrees threshold, occurred frequently in both patients later developing scoliosis as well as patients with milder or no scoliosis development. This study provided the umpteenth confirmation that structural scoliosis develops paired with the pubertal growth spurt [15, 16]. More specifically the present findings confirmed that the closure of the triradiate cartilage resulted as the best sign of pubertal spurt onset and scoliosis acceleration. Of course, in those affected by the most severe forms there is a larger acceleration in the Cobb angle during that period as compared to the milder forms. Out of 60 subjects that reached skeletal maturity, the prevalence of patients needing surgery was 3% with two subjects with curves of 69 and 73°. If we consider those exceeding 40 Cobb degrees the prevalence was 7%. Despite scoliosis in 22q.11.2DS has a prevalence around 50%, in the analyzed cohort the prevalence of severe cases quite low. This cohort included many subjects before scoliosis onset, this is more similar to a screening cohort, with lower prevalence of more severe cases. The early baseline (before onset), made it possible to predict scoliosis onset and to describe the trends of early phase of curve development. Low prevalence would require larger sample size and this is an encouragement to keep up monitoring these patients to develop better prediction in the future.

The tested predictors included age, gender, skeletal maturity parameters as well as commonly use 3D radiographic parameters for global balance and spinal alignment. Considering the overall performance of all the tested predictors we think that having also clinical parameters would have been helpful in discriminating patients with progressive scoliosis and those with self-resolving forms or with curves significantly below the diagnostic threshold. The most relevant clinical measures that may help in understanding onset of scoliosis progression are measures of axial rotation, such as the angle of trunk rotation ATR, as well as individual height and weight for individualized assessment of timing of the growth spurt. In the light of prediction modelling, we recommend to continue collecting clinical as well as radiographic data in 22q.11.2DS patients, over time, with regular and fixed time follow up to improve the dataset structure, the model performance and to minimize time bias. As the sample size increases and the number of measurement occasions increase, this will make it possible to test more parameters. Age is slightly correlated to skeletal maturity, in reality pubertal spurt happens at different age across subjects. The skeletal mature cohort include all stages of pubertal spurt, as it included subjects monitored from Risser 0 to Risser 3. In the univariate model the odds of having a maximum acceleration of Cobb angle significantly increase by 19% per year of age, but in the multivariate model age is not significant anymore. This may be due to an overfitting happening in the univariate model testing age and an underfit happening in the multivariate model because of stronger interactions with the triradiate cartilage closure factor, which in practice explains a larger proportion of the variance of the outcome than age.

The small sample may represent a study limitation. We limited it when we decided to include only subjects with Risser 0 at baseline, this has been done to make patients similar at baseline and remove subjects with a delayed diagnosis. We optimized the sample size by including in the skeletal mature cohort those with a minimum Risser score of 3. We choose the Risser 3 threshold because there is no significant growth from this stage on. Furthermore previously published paper applied the same criteria [19, 20]

We described a cohort of patients affected by 22q.11.2DS monitored prospectively over time for early detection of scoliosis onset, considering the high prevalence of this spine deformity in 22q.11.2DS population [2]. In this screened cohort it was possible to detect scoliosis at the earliest stages and this is the unique feature of the present research, attempting to explore the potential earliest predictors of scoliosis onset in those showing the most severe forms. The screening started earlier than usually done: mean age at start was around 8 years of age and included standard AP and lateral imaging [3, 17, 18]. More severe patients and patients with earlier diagnosis of 22q11.2DS, however, were monitored more or more frequently than less severe ones. This generates a confounder affecting the role of time in the model.

A comparison with the idiopathic scoliosis population would help in better understanding the difference in scoliosis evolution and growth with the 22q11.2DS population. Several studies have shown that the 22q11.2DS population has shorter stature at the end of their growth than the reference healthy population [10]. What implications this has on their chances of scoliosis progression, remains somewhat unsure. Joint laxity is typical of this population, but we could not investigate its role in the faster progressive group, “progressors” [8]. However, the present results are very similar to what was found in the idiopathic scoliosis population. For idiopathic scoliosis, it is still not possible to identify, before actual curve onset, subjects that will develop a structural curve, be it an aggressive form (progressors), or a more stable and less progressive form (non-progressors). The model performance was also similar to other studies testing Cobb prediction over time in the idiopathic scoliosis population based only on radiographic parameters [4, 15, 16, 18, 21]. Among the innovation brought by the developed model is the performance check of prognostic factors at baseline in the transverse and sagittal plane in the smaller sample that had reached the end of growth. None of the following baseline parameters: apex rotation, PITS values, the C7-SVA sagittal measure, C7 plumbline and CSVL in the coronal plane [11–14] resulted in a good discriminant of the more severe forms, nor a good predictor of curve magnitude progression.

The 22q.11.2DS subjects seem to go through a similar growth spurt, which is the period a scoliosis is most likely to develop and progress. Therefore, we confirm the similarity of the scoliosis history in the idiopathic and 22q.11.2DS populations.

## Conclusion

This study confirms the similarities in initiation and progression around the pubertal growth spurt between 22q11.2DS and AIS, and thus the applicability of this population as a model for biomechanical aspects of AIS. In the 22q.11.2DS population, many have spinal asymmetries at preadolescent age, and the true Cobb acceleration into a structural scoliosis happens when triradiate cartilage closes, that is the beginning of pubertal spurt. It is tempting to assume that this is similar in the AIS population but that cannot be concluded from this study. It is not possible to identify patients at risk for severe progression before the start of development of the deformity. The higher prevalence of scoliosis in 22q.11.2DS population provides samples which account for higher proportion of scoliosis subjects of different severity allowing to test different predictors. This is why we encourage the systematic collection of clinical and radiographic data from the moment of the 22q.11.2DS diagnosis. Regular follow-up is essential for early detection and necessary treatment to effectively prevent surgery for scoliosis in the 22q.11.2DS population. Regular follow-up will provide useful information to be able in the near future to accurately predict scoliosis onset and the moment of faster progression.

## Data Availability

The authors agreed on making the data available to any researcher requiring it to the corresponding author.

## References

[1] Negrini S, Donzelli S, Aulisa AG, Czaprowski D, Schreiber S, de Mauroy JC et al (2018) 2016 SOSORT guidelines: orthopaedic and rehabilitation treatment of idiopathic scoliosis during growth. Scoliosis Spinal Disord 13(1):3 29435499 10.1186/s13013-017-0145-8 PMC5795289

[2] Homans JF, Baldew VGM, Brink RC, Kruyt MC, Schlösser TPC, Houben ML et al (2019) Scoliosis in association with the 22q11.2 deletion syndrome: an observational study. Arch Dis Child 104(1):19–24 29627765 10.1136/archdischild-2018-314779

[3] Weinstein SL (1989) Adolescent idiopathic scoliosis: prevalence and natural history. Instr Course Lect 38:115–128 2649564

[4] Danielsson AJ (2013) Natural history of adolescent idiopathic scoliosis: a tool for guidance in decision of surgery of curves above 50°. J Child Orthop 7(1):37–41 24432057 10.1007/s11832-012-0462-7 PMC3566251

[5] Final Recommendation Statement: adolescent idiopathic scoliosis: screening | United States Preventive Services Taskforce [Internet]. Available from: https://www.uspreventiveservicestaskforce.org/uspstf/document/RecommendationStatementFinal/adolescent-idiopathic-scoliosis-screening. Accessed 25 Jul 2024

[6] Schlösser TPC, van Stralen M, Chu WCW, Lam TP, Ng BKW, Vincken KL et al (2016) Anterior overgrowth in primary curves, compensatory curves and junctional segments in adolescent idiopathic scoliosis. PLoS ONE 11(7):e0160267 27467745 10.1371/journal.pone.0160267 PMC4965023

[7] Janssen MMA, de Wilde RF, Kouwenhoven JWM, Castelein RM (2011) Experimental animal models in scoliosis research: a review of the literature. Spine J Off J North Am Spine Soc 11(4):347–358 10.1016/j.spinee.2011.03.010 21474088

[8] Homans JF (2018) Orthopaedic manifestations within the 22q11.2 deletion syndrome: a systematic review. Am J Med Genetics Part A. 10.1002/ajmg.a.38545 10.1002/ajmg.a.38545 29159873

[9] de Reuver S, Homans JF, Schlösser TPC, Houben ML, Deeney VFX, Crowley TB et al (2021) 22q11.2 Deletion syndrome as a human model for idiopathic scoliosis. J Clin Med 10(21):4823 34768342 10.3390/jcm10214823 PMC8584329

[10] Bassett AS, McDonald-McGinn DM, Devriendt K, Digilio MC, Goldenberg P, Habel A et al (2011) Practical guidelines for managing patients with 22q11.2 deletion syndrome. J Pediatr 159(2):332-339.e1 21570089 10.1016/j.jpeds.2011.02.039 PMC3197829

[11] de Reuver S, Homans JF, Schlosser T, Pasha S, Kruyt MC, Castelein RM (2021) Variations in the sagittal plane precede the development of scoliosis: a proof of concept. Stud Health Technol Inform 28(280):18–22 10.3233/SHTI210426 34190054

[12] Castelein RM, Pasha S, Cheng JC, Dubousset J (2020) Idiopathic scoliosis as a rotatory decompensation of the spine. J Bone Miner Res Off J Am Soc Bone Miner Res 35(10):1850–1857 10.1002/jbmr.4137 32697856

[13] Pasha S, de Reuver S, Homans JF, Castelein RM (2021) Sagittal curvature of the spine as a predictor of the pediatric spinal deformity development. Spine Deform 9(4):923–932 33449344 10.1007/s43390-020-00279-y

[14] Schlösser TPC, Simony A, Gerdhem P, Andersen MØ, Castelein RM, Kempen DHR (2021) The heritability of coronal and sagittal phenotype in idiopathic scoliosis: a report of 12 monozygotic twin pairs. Spine Deform 9(1):51–55 32761476 10.1007/s43390-020-00172-8 PMC7775859

[15] Lonstein JE, Carlson JM (1984) The prediction of curve progression in untreated idiopathic scoliosis during growth. J Bone Joint Surg Am 66(7):1061–1071 6480635

[16] Parent EC, Donzelli S, Yaskina M, Negrini A, Rebagliati G, Cordani C et al (2023) Prediction of future curve angle using prior radiographs in previously untreated idiopathic scoliosis: natural history from age 6 to after the end of growth (SOSORT 2022 award winner). Eur Spine J 32:2171–2184 37059884 10.1007/s00586-023-07681-w

[17] Di Felice F, Zaina F, Donzelli S, Negrini S (2018) The natural history of idiopathic scoliosis during growth: a meta-analysis. Am J Phys Med Rehabil 97(5):346–356 29493563 10.1097/PHM.0000000000000861

[18] Dolan LA, Weinstein SL, Dobbs MB, Flynn JMJ, Green DW, Halsey MF et al (2024) BrAIST-calc: prediction of individualized benefit from bracing for adolescent idiopathic scoliosis. Spine 49(3):147–156 37994691 10.1097/BRS.0000000000004879 PMC10841822

[19] Negrini S, Donzelli S, Negrini F, Arienti C, Zaina F, Peers K (2021) A pragmatic benchmarking study of an evidence-based personalised approach in 1938 adolescents with high-risk idiopathic scoliosis. J Clin Med 10(21):5020 34768544 10.3390/jcm10215020 PMC8584294

[20] Dolan LA, Donzelli S, Zaina F, Weinstein SL, Negrini S (2020) Adolescent idiopathic scoliosis bracing success is influenced by time in brace: comparative effectiveness analysis of BrAIST and ISICO cohorts. Spine 45(17):1193–1199 32205704 10.1097/BRS.0000000000003506

[21] Negrini F, Cina A, Ferrario I, Zaina F, Donzelli S, Galbusera F et al (2023) Developing a new tool for scoliosis screening in a tertiary specialistic setting using artificial intelligence: a retrospective study on 10,813 patients: 2023 SOSORT award winner. Eur Spine J Off Publ Eur Spine Soc Eur Spinal Deform Soc Eur Sect Cerv Spine Res Soc 32(11):3836–3845 10.1007/s00586-023-07892-1 37650978

