# Correction: What can we learn from scoliosis in children with the 22q11.2 deletion syndrome? Prognostic factors at pre-adolescent age for fast progressive, mild and self-resolving forms during adolescence

**DOI:** 10.1007/s43390-025-01155-3

**Published:** 2025-08-27

**Authors:** Sabrina Donzelli, Peter Lafranca, Maarten van Smeden, René Castelein, Tom Schlösser

**Affiliations:** 1 Istituto Auxologico Italiano, Milan, Italy; 2 Department of Orthopaedic Surgery, UMC Utrecht, Utrecht, The Netherlands; 3 Julius Center for Health Sciences and Primary Care, Utrecht, The Netherlands

**Correction: Spine Deformity (2025) 13:1179–1187** 10.1007/s43390-025-01073-4

In this article the author’s name Maarten van Smeden was incorrectly written as Marteen Van Smeden.

The original article has been corrected.

